# Strengthening vaccine uptake: a qualitative assessment of community health worker educational resource needs and community perspectives on vaccination in Western Kenya

**DOI:** 10.3389/fpubh.2025.1661069

**Published:** 2025-11-14

**Authors:** Nophiwe Job, Sei-kashe M’pfunya, Sandra Mudhune, Benson Nyawade, George Omondi, Mumma Edelquinn, Moses Sadia, Erick K. Odhiambo, Xian Ho, Nadine Skinner, Jamie Sewan Johnston, Victoria Ward, Jane Wamae

**Affiliations:** 1 Stanford Center for Health Education, Cape Town, South Africa; 2 Lwala Community Alliance, Rongo, Kenya; 3 Health Department, County Government of Migori, Migori, Kenya; 4 Dimagi, Inc., Cambridge, MA, United States; 5 Stanford Center for Health Education, Stanford University, Stanford, CA, United States; 6 Department of Pediatrics, Stanford University School of Medicine, Stanford, CA, United States

**Keywords:** community health workers, digital training, vaccine education, digital divide, malaria vaccine, community perspectives, CHW training

## Abstract

**Introduction:**

Vaccination strengthens health systems by preventing the spread of infectious diseases and reducing morbidity and mortality. The introduction of the malaria vaccine in Kenya, alongside the growing access to technological tools, offers a timely opportunity to explore the educational needs of community health workers (CHWs) and the feasibility of digital training and health education resources for CHWs.

**Methods:**

A qualitative descriptive study was conducted among CHWs and community members in two sub-counties in Migori County. In-depth interviews were held with 20 CHWs selected through stratified random sampling from 72 community health units grouped into eight strata. Additionally, four focus group discussions were conducted with 32 community members. Thematic analysis was conducted using both inductive and deductive coding approaches.

**Results:**

CHWs strongly preferred in-person training with visual aids but showed cautious interest in digital learning tools. Key barriers to digital training include limited digital literacy, language constraints, smartphone access, and associated costs. Community members supported vaccination and trusted CHWs due to their training and affiliation with health facilities. However, vaccine hesitancy persists, driven by fears of side effects, cultural beliefs, and misinformation. Confusion surrounding the limited geographic rollout of the malaria vaccine has contributed to skepticism, with some misinformation linking the vaccine to family planning and other health risks.

**Discussion:**

A comprehensive, community-centred communication strategy addressing the geographic rollout of the malaria vaccine is necessary. Overcoming the ‘digital divide’ through targeted training, improved technology infrastructure, and user-friendly technology may enhance CHWs’ capacity to deliver effective vaccine education within communities.

## Introduction

Effective delivery of vaccine education, which is essential for improving outcomes in community health, depends on sufficient training of community health workers (CHWs). Global public health actors now acknowledge the potential of innovative digital education resources to better equip the routine practices of CHWs. Immunisation is a critical public health strategy to decrease global child mortality from infectious diseases (1). Since the enactment of Immunisation Agenda 2030, more than 20 life-threatening diseases can be prevented with vaccination (2–4). This is emphasised by the worldwide reduction of deaths due to vaccine-preventable diseases from 12.5 to 5.3 million from 1990 to 2018 (73). However, in 2023, there were 14.5 million children who had not received *any* vaccinations, so-called ‘zero-dose’ children, across the world (4, 5). The global burden of low vaccination coverage and zero-dose children is significant in low- and middle-income countries (LMICs), particularly in Sub-Saharan Africa (SSA) (6, 7, 74).

In Kenya, overall vaccine coverage ranged from 2 to 95%, while only 4 out of 9 (44%) vaccines in the national immunisation schedule achieved 90% or higher coverage in 2013 (4, 72). In 2023, overall vaccine coverage ranged from 6 to 97%, while 9 out of 13 (69%) vaccines in the revised schedule achieved 90% or higher coverage (4, 72). In southwestern Kenya’s Nyatike and Awendo areas, immunisation rates in the last quarter of 2023 were 93 and 87%, respectively (8). The recent roll-out of the malaria vaccine in Kenya presents a timely opportunity to explore both community and CHWs’ perspectives on the new vaccine and to assess the educational needs of CHWs to deliver vaccine education effectively. This study also aimed to identify the current barriers to childhood vaccine uptake and explore accessibility and potential use of digital tools by CHWs and community members to support the distribution of vaccine-related information.

Community perspectives about vaccines are formed by cultural and religious beliefs, often resulting in some communities rejecting immunisation due to misconceptions or mistrust. Some religious groups prohibit the use of conventional medicine, including vaccination in some settings (9–11). In some cultures and religious beliefs, it is believed that vaccines lead to death, as it is believed that children are born immune to illness. Thus, in most cases, traditional medicines were more trusted for managing childhood illnesses (10). A qualitative study was conducted among national and county-level immunisation officials and caregivers in four counties in Kenya to explore the factors contributing to vaccine hesitancy. The study highlighted that in the case of both routine immunisation (RI) and the Human Papilloma Virus (HPV) vaccine, insufficient knowledge and understanding of how vaccines work lead to hesitancy, and thus to missed opportunities for immunisation (9). Caregivers expressed concern regarding taking children for RI out of fear of side effects such as swelling, while some caregivers assumed that RI was only for sick children. Cultural beliefs and misinformation about the HPV vaccine led to hesitancy, particularly in rural areas, as the vaccine was perceived as a form of contraception and feared for encouraging promiscuity among adolescent girls (9, 12–15). Higher education levels were correlated with higher vaccine uptake and understanding of the importance of vaccines (9, 10, 14).

Access barriers also significantly hinder vaccine uptake. Poverty and competing household priorities may contribute to lower vaccination rates since, while vaccines are provided free of charge, indirect costs such as transportation to health facilities and potential income loss from taking time off work can be prohibitive for low-income families (9, 11, 16). Due to Kenya’s vast geographical diversity, reaching remote or marginalised populations requires additional resources, and the lack of transportation and infrastructure in these areas can further reduce vaccine uptake (9). Rural areas face greater challenges, such as fewer healthcare facilities, longer travel distances to vaccination centres, and fewer healthcare workers available to deliver immunisation services (17). This discrepancy contributes to low overall demand for vaccines in rural communities. Other known factors driving low demand for vaccines among Kenyan communities include health system challenges such as supply chain issues leading to vaccine shortages, healthcare worker strikes and high health worker-to-patient ratios (9, 10, 17). These health system challenges can also erode public trust in the healthcare system, leading to a reduction in the community’s confidence in health initiatives such as vaccination (9, 10, 17). Lack of trust in the government and society is a key predictor of vaccine hesitancy (5, 18).

While existing literature underscores the effectiveness of digital tools for health education, research into the integration of these resources into the routine practices of CHWs remains limited. CHWs serve as trusted sources for enhancing equity in vaccine access, particularly for under-immunised and zero-dose children (19–23). Traditionally, CHWs engage in face-to-face interactions, which can be both time-consuming and geographically restrictive and universal access to health through digital means is a growing possibility (24–26).

Digital platforms, such as WhatsApp, are increasingly employed to disseminate vaccine-related information, significantly influencing uptake among specific demographics, including pregnant women (27). As mobile phone penetration and internet connectivity increase across Africa, digital health strategies may offer a cost-effective means for disseminating vaccine information through CHW outreach (25, 28–30). Supporting CHWs with digital tools may facilitate scalable and impactful vaccine education, facilitate real-time data collection on vaccination rates and community health trends, providing real-time updates on vaccine safety and schedules while ensuring the dissemination of accurate information through refresher courses for CHWs (31–36). Interactive content, including videos and chatbot-assisted messaging, can enhance engagement and comprehension among diverse literacy audiences (31, 33). Importantly, digital training programmes must be directed at both CHWs and the general public to ensure the effective use of these digital health tools (35).

## Methodology

### Study design

This paper presents a descriptive qualitative study that employs in-depth interviews (IDIs) with CHWs and focus group discussions (FGDs) with community members. The study aims to explore the potential for using digital tools to address the educational needs of CHWs in delivering effective vaccine education. Specifically, it seeks to identify common misconceptions regarding vaccinations and to assess the access to and utilisation of technology, including social media platforms and smartphone ownership, among both CHWs and community members. Because the study was initiated as the first phase in developing educational training materials for CHWs to promote vaccine knowledge and acceptance, prototype educational material was also shared with participants to gauge perceived usefulness and effectiveness in meeting their needs. Descriptive qualitative analysis allows for deep exploration of a phenomenon, capturing the nuances of participants’ experiences, perceptions, and behaviours (37, 38). This method also prioritises the voices and perspectives of participants, ensuring that findings reflect their lived realities rather than researcher-imposed ideas (39, 40). This helps researchers understand complex social, cultural, or health-related issues that quantitative studies may overlook, such as ‘intention to and/or willingness to vaccinate’ and ‘intention to and/or willingness to use digital tools’.

### Sampling

A total of 20 CHWs were selected through stratified random sampling to participate in IDIs across Nyatike and Awendo sub-counties located in Migori County’s rural western Kenya. The sample was stratified into eight groups, consisting of a total of 72 Community Health Units (CHUs) across the two sub-counties. In each group, the 20 CHWs were randomly selected from a pool of 846 based on access to a healthcare facility and gender. Recruitment of the CHWs followed a predetermined method that allowed for a minimum of 12 participants from every CHU before reaching a capped number of interviews. A total of 13 female CHWs and seven male CHWs were included in the study. The research study also included four FGDs, which included *n* = 32 randomly selected 32 community members across both sub-counties who were older than 18 years, male and female, parents who were being served by the CHWs.

### Data collection materials

**Figure 1:**
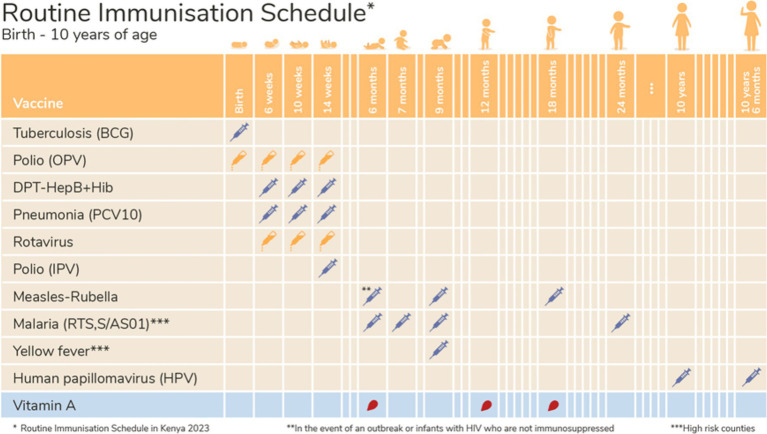
Prototype material 1.

**Figure 2:**
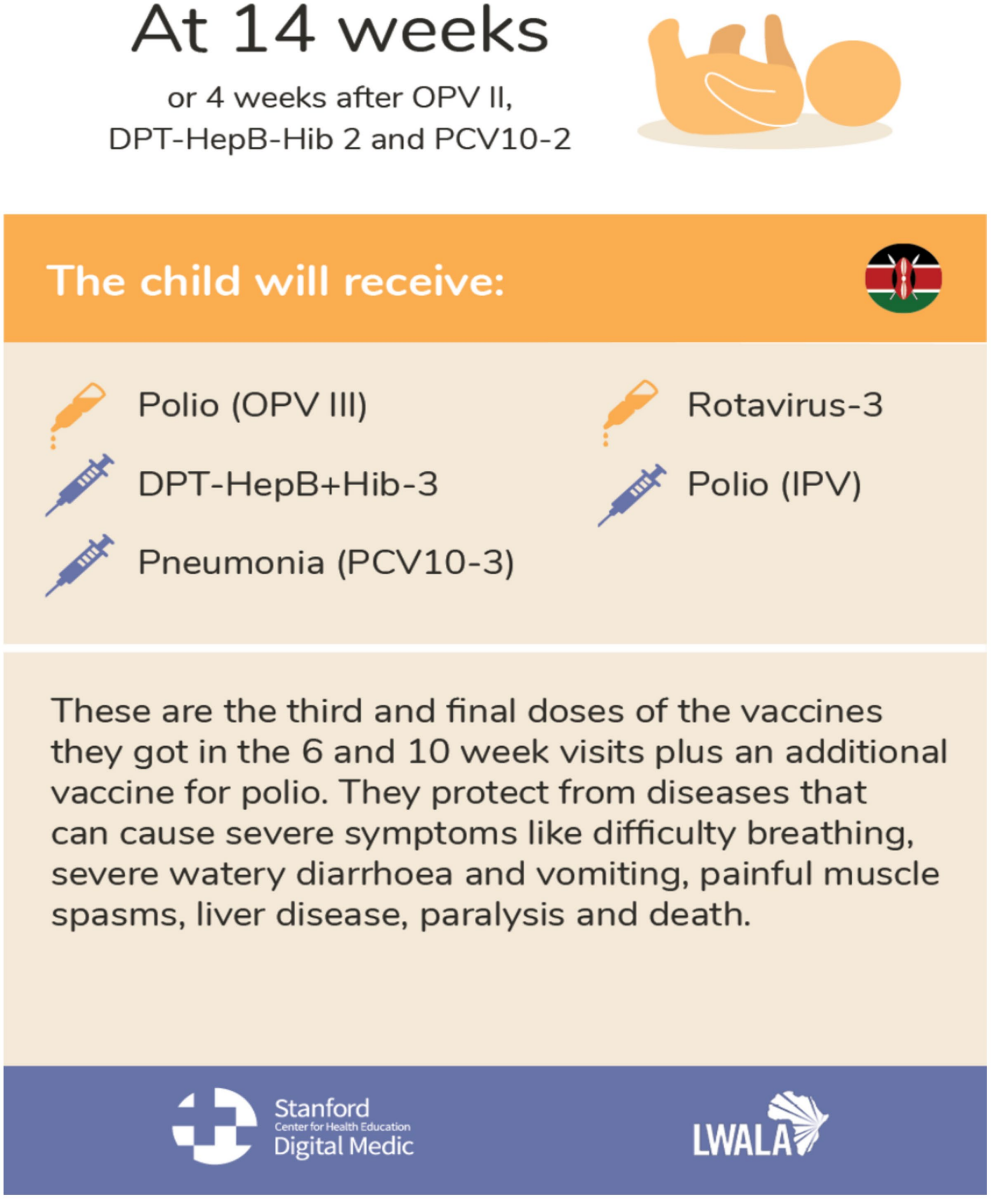
Prototype material 2.

A semi-structured interview guide was developed for conducting IDIs among CHWs (Appendix A). The guide included open-ended questions to allow the emergence of ideas and themes that may not have been anticipated during the discussion, while providing a framework to ensure that all key research questions are covered (41, 42). The interview guide covered questions concerning CHW sources of vaccine information, CHW training needs, educational resources required by CHWs, reflections on educational materials (see Figures 1, 2), perceived sources of vaccine hesitancy in their community, perceived uptake and acceptance of vaccines (including regarding the new malaria vaccine) and perceived smartphone access and use among themselves and their communities. Lwala researchers conducted the IDIs in English and Dholuo, as preferred by participants. CHWs were allowed to ask questions about the study before consenting to participate, and an electronic copy of the consent form was shared with them for their records. The IDI’s duration was ±60 min.

A flexible and adaptive semi-structured FGD guide (Appendix B) was developed to investigate the sources of hesitancy or challenges to vaccination for families in the community. The guide included open-ended questions to uncover the ‘why’ behind behaviours and decisions made by community members concerning RI and vaccination (43). The discussion guide covered questions about the community’s sources of information, preferred communication methods, knowledge and attitudes towards RI and the new malaria vaccine, their access to technology such as smartphones and their use of online platforms.

The dynamic FGD setting allowed participants to build on each other’s ideas, leading to richer and more diverse discussions that may not have emerged in individual interviews (41). The setting also allowed researchers to uncover shared norms, values, and collective attitudes within the community, which is valuable information for creating contextually sound interventions (41, 44, 45). The discussions were facilitated in person, in English, and in Dholuo by experienced Lwala researchers, and community members were allowed to ask questions about the study before consenting to participate. The duration of the FGD was ±60 min.

### Data analysis

All IDIs and FGDs were audio-recorded. To prepare and organise the data, the audio files of all interviews and FGDs were transcribed internally (clean verbatim transcription) into Microsoft Word documents and then translated into English for analysis. The documents were imported into *Dedoose*, a web-based application for organising and analysing qualitative research.

As the study examined a complex issue such as vaccine hesitancy, thematic analysis was conducted using deductive and inductive approaches. This enabled the researchers to apply predefined codes or definitions derived from vaccination literature and the study objectives while allowing patterns and themes to emerge naturally, without being constrained by an external theory or framework (41). Researchers discussed the literature on vaccine hesitancy, and a codebook was created with predefined codes or themes from the literature. The researchers thoroughly reviewed the transcripts to gain familiarity with the data and identified initial ideas emerging in the data. Each transcript was independently coded by two researchers. The data was segmented and coded according to the predefined categories. Where new codes or themes emerged from the data, the researchers compared responses across participants to refine their understanding of each code and ensure that the themes were consistently applied. Through this iterative process of coding and comparison, the researchers identified patterns and recurring ideas in the data. The final findings were interpreted in relation to the original study research questions (39).

### Ethics approval

The study was a collaboration between Stanford University’s Digital Medic researchers and Lwala Community Health Alliance in Kenya. Local approval was obtained from Strathmore University Institutional Scientific and Ethical Review Committee (SU-ISERC1715/23), the Kenyan national research governing body, the National Commission for Science, Technology, and Innovation (NACOSTI/P/23/26284) and Stanford University (eProtocol #69793). Informed consent was obtained from CHWs and community members, ensuring they understood their rights, including the right to withdraw at any time. Personal identifiers were not recorded in the study documentation, and any quotes or references to participants were deidentified. FGDs were conducted in private setting, group sizes were kept small, participants were reminded of the confidentiality of their contributions and encouraged to refrain from discussing the content of the discussions with others outside the group.

## Results

The results present the findings from a total of 24 transcripts from 52 respondents (20 CHW IDIs and 32 community stakeholders in four FGDs). With the recent introduction of the malaria vaccine, the study sought to explore community perspectives on the malaria vaccine and other RI while assessing CHWs’ educational needs for effective vaccine education delivery. Eight (8) primary themes emerged from the analysis: (1) Community knowledge and concerns regarding vaccination, (2) Community hesitancy towards the malaria vaccine, (3) Community preference for in-person vaccine information delivery and curiosity about digital platforms, (4) Community technology use and access challenges posed by the digital divide, (5) CHWs positive attitudes towards vaccines and need for continued training, (6) CHW preferences for receiving training through in-person and visual modalities, (7) CHW preferences for in-person information sharing modalities with visual aids, (8) Positive CHW prototype education material feedback.

### Community knowledge and concerns regarding vaccination

In discussions with community members, it was apparent that the participants generally understood the benefits of vaccination to include the decreased risk of contracting and/or exacerbating illnesses, and many participants were able to name a few vaccines they were familiar with. Some participants trusted vaccines completely for the healthy growth and development of children. Participants also deemed vaccinating children on time (according to the immunisation schedule) as important. Some respondents reported being familiar with specific vaccinations (e.g., polio, tetanus, rotavirus, BCG, malaria) while others reported familiarity with the time at which vaccinations should be administered (e.g., 7 months, 9 months). Generally, all the groups portrayed positive attitudes towards vaccines, as demonstrated in the following quotes:

*‘Even if the child gets sick, I can still have a little peace because they have taken all the required vaccines.’—FGD 1.*

*‘Child grows very healthy and strong after receiving vaccination.’—FGD 3.*

*‘What encourages me to go for vaccination is that I do not want to backdate defaulted vaccines.’—FGD 1.*

Even though there was a positive attitude towards vaccination among the groups, the participants were aware of the challenges and negative attitudes that still prevail in their communities regarding vaccinating children. They discussed the existence of families who were still reluctant to vaccinate their children. The reasons cited for families not vaccinating their children can be categorised into two groups: personal/family attitudes and beliefs, and structural barriers. The groups acknowledged that *‘Not all believe in science.’ (FGD 4)*, explaining that some families did not trust modern medicine or science and thus preferred traditional medicine or herbal remedies for caring for children. They acknowledged that, in some cases, family members may not share the same perspectives on vaccination, often leading to conflict within the household. The quote below from one of the FGDs demonstrates this.

*‘In the household, we have different views. It could be the husband had more information than the wife. So at times, the mother does not see the need to take the child but the father does. So this brings conflict.’—FGD 4.*

One participant shared their story about secretly taking a child for immunisation after being instructed not to by other family members. Stating that, *‘at times the directives will not allow you to take your child for vaccination. But from my end if I see date for vaccination is passing, I will hide and take the child for vaccination secretly’ (FGD 1).* It appeared to be a common phenomenon as it was again discussed in a different FGD stating that *‘…more educated mothers will secretly take the child for vaccination without the knowledge of the father.’ (FGD 4).*

In discussions about responsibility for childhood vaccination, community members largely felt that women, particularly mothers, took the lead in ensuring children and other relatives were taken to health facilities. Some community members believed that both parents should share the responsibility, including financial responsibility, of supporting their children’s vaccinations and healthcare visits. They concurred on the importance of men taking a more active role but acknowledged the need for greater education efforts for men in their community to fully understand and appreciate vaccination. A man in one of the FGDs stated:

*‘Yes, I fear because I do not know much about vaccination and question myself how did our grandparents survive without vaccines. So, at times, we as men, if we are not taught then we will fear taking children for vaccination. And if there are any side effects you should also let us know. Because we as men can tell our wives not to take children for vaccination.’—FGD 1.*

Another belief associated with vaccine hesitancy was religion. The groups discussed that some families ‘*believe it is God who protects the child and therefore, they do not take them to hospital.’ (FGD 1)*, that *‘prayers prevent the diseases. “Jesus is Enough”’ (FGD 2)* and that *‘vaccinating children frequently lowers their immune system.’ (FGD 1)*.

Other beliefs included myths and misinformation that vaccination caused children to be reproductively challenged in adulthood, caused children to have learning difficulties and that the vaccines weakened the immune system of children and even cause paralysis, as demonstrated by these quotes:

*‘… Some parents believe that…the children are being vaccinated to prevent them from giving birth in the future.’—FGD 4.*

*‘Vaccination will paralyse the child.’—FGD 2.*

*‘… We were told that children who get vaccinated do not become intelligent.’—FGD 1.*

Finally, another personal barrier to vaccination was the belief that vaccination caused pain for children, made them sick and resulted in sleepless nights for caregivers. This is demonstrated by the quote below:

*‘The challenge that we have is when you take your child out for immunisation. When you come back to the house you would not be able to sleep because the child will constantly cry in pain.’—FGD 4.*

The groups also discussed structural barriers to vaccination. The barriers included distance from the health facility, lack of money for transport to health facilities, vaccine stock shortages at facilities and long queues at health facilities. Participants explained that people often lived far from health facilities and usually needed to travel under bad weather conditions to make it to facilities. Often, caregivers arrived too late at facilities and would be asked to return the following day. In some instances, caregivers require money to be able to travel to facilities and thus can only make a limited number of trips for vaccinations. Sometimes, when caregivers arrived at health facilities, there would be long queues, requiring caregivers to spend the whole day at the facility instead of being at work. In addition, caregivers often reported a lack of vaccine stock on days when they are at the facility and therefore see the task as a waste of time. Some of these barriers are demonstrated in the following quotes:

*‘I am staying far from the hospital hence reaching there very late after the clinic time and being sent away to visit the clinic the following day.’—FGD 1.*

*‘I found that there were no drugs and hospital is too far from my place.’—FGD 3.*

Another barrier to vaccination was the fear of being reprimanded by nurses when caregivers missed vaccination appointments. This fear often discouraged caregivers from visiting health facilities altogether, even for reporting other childhood illnesses, stating that mothers are fearful of *‘the embarrassment of being yelled at by the healthcare providers.’ (FGD 4)*. Participants described nurses’ behaviour as *“rude”* and *“judgmental,”* leading to caregiver embarrassment and, in some cases, continued non-compliance. When catch-up doses were administered, some caregivers mistakenly believed their children were being ‘*overdosed*’ (FGD 2), a misconception that could have been addressed through better communication with healthcare providers. The following quotes illustrate these challenges:

*‘The nurses at the hospital should also learn to speak to us with respect… There are some who use harsh tone on us… that discourages a lot of people.’ FGD 1.*

*‘I have witnessed the healthcare provider being rude to the mother who came late and her mother’s booklet was thrown back to her. She swore not to return to the facility again.’—FGD 4.*

### Community hesitancy towards the malaria vaccine

When explicitly asked about malaria, community members had varying levels of awareness and understanding of the vaccine. There was a well-informed group that was aware of the malaria vaccine, its benefits for children under the age of five, and the risks associated with not vaccinating. They expressed no hesitation in ensuring their children received the vaccine, as illustrated in this quote:

*‘I do not have any reservations about vaccines. For example, when a child gets malaria vaccine it is able to protect the child from getting severe malaria.’—FGD 1.*

There were also some misinformed or skeptical community members who had limited access to accurate information and misconceptions about the malaria vaccine. Some believed it was a form of family planning, conflicted with religious beliefs, or could harm a child’s growth, potentially causing fever or even death. Additionally, some did not understand how the necessity of the vaccine varied by geographic location. A CHW explains why some of these concerns exist in an IDI:

*‘When the [Malaria] vaccine first came it was during that same time the young girls between 9–15 years were being vaccinated for HPV. Part of the community believed that as the young girls were being vaccinated for family planning, so the malaria vaccine was being used to prevent their children from giving birth… They are asking why it [Malaria vaccine] is only in Migori and if you go to Nairobi it is not there. Some say that when they come from Nairobi and back to the village we say that we have to vaccinate them. So, when they ask we tell them that the level of malaria in Nairobi is different from that in Migori.—IDI 7.*

Finally, there was an unaware or curious group of community members who had never heard of the malaria vaccine and had several questions, including whether malaria could recur after vaccination, why the vaccine had not existed before, why it was now considered important, the age eligibility criteria, and how the vaccine was administered. Some IDIs with CHWs show the curiosity of community members regarding the Malaria vaccine:

*‘They ask reasons why their children are being vaccinated and yet they already have mosquito nets and their houses were sprayed?’—IDI 13.*

*‘Will vaccination against malaria will not interfere with a child’s growth?*

*If a child does not get the second shot, can he get the third?’—IDI 12.*

*‘They ask about types of malaria. They ask how the malaria vaccine works and why it is administered that way.’—IDI 17.*

### Community preference for in-person vaccine information delivery and curiosity about digital platforms

Community members strongly expressed a preference for receiving in-person vaccine information from their health facilities and from CHWs during household visits. They had confidence in the information shared by CHWs as they were seen as trained sources from health facilities. They had even more confidence in the CHWs if they were accompanied by another health professional from their health facilities. Ultimately, the community preferred in-person communication to any other form of engagement. This is illustrated in the quotes below:

*‘I trust my CHW so much, when you bring me information I accept because I know it is most likely from the hospital.’—FGD 1.*

*‘… They are knowledgeable since they are working hand in hand with the doctors.’—FGD 3.*

The community also preferred and trusted information shared in-person in communal meeting environments such as bars or spaces where people drink, churches, ‘*the village Chief’s Barraza’*, gatherings at the market, Ministry of Health outreaches and campaigns, village elders’ announcements and schools. Beyond in-person communication, community members also preferred receiving information through traditional media, i.e., television, radio, billboards, and/or posters. Discussions revealed that traditional media had been commonly used to share health information before the emergence of online social media, making it a trusted source. The groups did acknowledge that some families still do not have access to traditional media like television and radio, even though they consider it a credible source, highlighting the importance and preference for in-person communication methods in their communities. A community member explained in the quote below:

*‘I agree with all that has been said. However, most people do not have TV and Radio. The CHW in my area usually announce walking around in the community.’—FGD 1.*

In some groups, some community members expressed either existing use and/or interest in using digital solutions for vaccine education, noting a liking to mobile SMS or online content as useful avenues to receive information. The main deterrent to obtaining health information from online platforms was the cost of mobile data and smartphone use and access. Furthermore, some of the participants had great distrust for receiving health information through social media platforms, illustrated in the quote below:

*‘I do not trust such messages because there are many scrupulous people on the internet. But when the chief announces, I trust that because it is from the government.”—FGD 1.*

### Community technology use and access challenges posed by the digital divide

Community members widely acknowledged a significant increase in smartphone ownership and usage within their communities, a trend partly attributed to the availability of local credit facilities such as ‘*M-KOPA*’. M-KOPA is a UK-headquartered fintech company operating in several African countries, including Kenya, Uganda, Nigeria, Ghana, and South Africa. It provides underbanked customers with affordable smartphones and digital financial services through a pay-as-you-go model (46). Having said that, the community noted that smartphone access was not evenly distributed across the population, with younger individuals being more likely to own and use these devices compared to older adults, as illustrated in the following quotes:

*‘These days I see a lot of people have smartphones. Because we have “M-KOPA” phones where an individual will buy a phone on hire-purchase and pay slowly.’—FGD 4.*

*‘If am to rate it out of ten, eight out of every ten smartphone users are the youth. The adult population has the least ownership of smartphones.’—FGD 4.*

Participants noted that despite the growing prevalence of smartphone ownership in their communities, digital literacy remains a considerable challenge among users. Many users struggled with device navigation and lacked a complete understanding of smartphone functionality. Language barriers were also identified as a key obstacle to effective smartphone use. Additionally, financial and infrastructural constraints, including the high cost of data bundles and poor network coverage, were cited as significant limitations. Lastly, the theft risk was highlighted as a deterrent to smartphone ownership in certain communities. The quotes below illustrate a few challenges unpinning the digital divide that were mentioned in FGD 4:

*‘In order to access digital information, you may need an email address. This is intensive to sign up. So I do not have the skills to navigate through these kinds of features.’—FGD 4.*

*‘You’ll find individuals who have smartphones just take photos whenever they are out on a safari but cannot really navigate through it.’—FGD 4.*

*‘There are individuals who have smartphones but would not be able to understand the language. An example they would want to create a Facebook account but would be challenged.’—FGD 4.*

*‘Many smartphones mainly use English and at times I may be illiterate. I may have money but the language used by the smartphone will be a huge challenge. I have seen individuals going to withdraw cash from “M-PESA” and ask the customer desk to help them withdraw money. Meaning they have no idea of how to navigate through some apps on their phones.’—FGD 4.*

*‘One reason that can limit individuals from buying smartphones is that people see smartphones as fragile and requiring an extra layer of protection. Another thing is that smartphones attract thieves and can be easily stolen, unlike feature phones.’—FGD 4.*

The community demonstrated familiarity with sharing and receiving health information through online social platforms, including WhatsApp, Facebook, Instagram, TikTok, Snapchat, and Twitter. They also expressed a willingness to utilise these platforms for health information exchange. However, their ability to do so was contingent on their prior familiarity and ability to access such platforms. Many respondents identified a diverse range of online platforms and acknowledged the potential for misinformation to spread on social media, as illustrated by the quote below:

*‘There are individuals who seem to be possessed. They can sit down and cook up something and loads it to your group and this can spark debates. So before I share information to the groups I have to verify if these pieces of information are true. Because I might send something which is a rumor.’—FGD 4.*

### CHWs positive attitudes towards vaccines and need for continued training

Among the CHWs interviewed, most felt that they knew the importance of vaccination and had a basic understanding of how vaccines work, the different types, the benefits, and the side effects, particularly due to the training they received. They expressed reliance on being trained and the importance of being knowledgeable before visiting households for any health activities.

*‘… So even when I’m giving someone advice, I do it from the trainings I’ve received.’—IDI 2.*

*‘When you are going to visit people, you must be more knowledgeable. When you are not knowledgeable then you will not be respected… It is upon me as the CHW to give the right information…’—IDI 7.*

In terms of their attitudes, the CHWs interviewed were pro-vaccines. They had trust that vaccines work. In addition, they understood vaccine side effects, which did not prevent them from confidently advocating for vaccines in their communities. A CHW in IDI 20 said, *‘I do advocate the benefits of vaccination to them, and this removes any negative perception about vaccination’*. Most CHWs gained vaccine confidence from training, as shown in the preceding quotes, and from experience serving their communities and seeing behaviour change and positive results thereafter. Some also noted that vaccination uptake was more widely accepted when they ‘*set an example by taking the vaccine’* (IDI 9) themselves, making it an effective approach to conveying its importance.

Most CHWs felt that they had received adequate training on vaccines. The source of this training was Lwala and/or the Ministry of Health, with ongoing in-service training usually totalling 6–7 h per month. The format of prior training was primarily in-person, and some refresher training was conducted during immunisation campaigns following outbreaks of some vaccine-preventable diseases. Most CHWs reported that they gained useful information from these training sessions and felt that these opportunities were the source of their confidence in supporting their households. There were a few newly recruited CHWs who had not been trained at all and felt that they did not have enough vaccine knowledge to impart to community members. However, CHWs felt that any gaps in vaccine knowledge could be filled through additional training or learning from their existing resources, like their Mother and Children booklet, which bears information related to pregnancy, nutrition and early childhood development, their CHA, local facility or from Lwala. This is illustrated in the quotes below:

*‘If I am taken through training, I will be able to answer. But for now I only use the little knowledge I have from mother child booklet.’—IDI 9.*

*‘When I do not have responses I call my CHA, and we can always go back together to that household to respond to that question. So any difficult question that I get I call my CHA.’—IDI 3.*

### CHW preferences for receiving training through in-person and visual modalities

When asked to identify their preferred mode for receiving health information and training, the CHWs interviewed identified in-person training sessions, particularly those incorporating visual projections, as highly valued, as they provided interactive and engaging learning experiences. CHWs frequently consulted their CHAs and health professionals at local health facilities for guidance and clarification. Training materials, including the Mother and Child Booklet, charts, job aids, and other reference books, were also commonly used as reliable sources of information. Furthermore, CHWs expressed openness to digital training methods, recognising the convenience of accessing educational resources on their mobile phones at all times. A few CHWs expressed reluctance towards using technology due to apprehensions about making errors on their work devices. CHWs’ desire for visual aids during training is expressed in the following quotes:

*‘If anything comes I’ll embrace it because any new thing that comes, comes with ease. You see back then before the phones came, everything we did we used to write in papers. Maybe if they put it [New training materials] in my phone it can be nice.’—IDI 5*.

*‘… Books and the person teaching me can use videos to show how it is done practically.’—IDI 9.*

### CHW preferences for in-person information sharing modalities with visual aids

CHW had varied preferences for sharing information; however, they predominantly preferred in-person communication, including community gatherings and dialogues, and door-to-door household visits. These methods enabled them to educate communities and disseminate crucial information about vaccinations effectively. Some CHWs preferred using various visual aids to enhance their communication, such as referencing the Mother and Child booklet, utilising job aids or charts stating that ‘*that chart has pictures and it is able to give me detailed information of what I’m training in the household. When I’m training and someone is able to visualise that through the charts. That is one of the ways that I use to promote vaccine intake.’ (IDI 2)*. They also mentioned the desire to have images and/or videos to show to clients from their phones ‘*for people to watch the explanation.’ (IDI 9)*. Generally, CHWs appreciate visual aids in any format. *‘If I have pictures in books or even videos it will be easier rather than just reading to them. What they can see is better.’ (IDI 4).*

Findings from the in-depth interviews revealed that the primary topics CHWs discuss with communities focus on maternal and child health, responding to disease outbreaks as they arise, and addressing vaccine-related myths and misinformation. Therefore, CHWs dedicate most of their time to caring for mothers, pregnant women, and children under the age of five. Their interactions often begin with verifying whether childhood immunisation schedules are up to date and encouraging full vaccination. CHWs frequently reported the need to reiterate the benefits of vaccination and emphasise the importance of ensuring that children receive all necessary vaccines. Additionally, discussions on other vaccines were primarily influenced by ongoing outbreaks, with CHWs educating communities on the significance of immunisation against the prevailing disease threat. Lastly, their communication efforts included dispelling myths and misinformation, particularly concerns regarding illness and potential side effects associated with vaccination, as illustrated in this quote:

*‘What I usually try to do when I do the household visits…Again, sometimes with the challenges that they get after vaccine injection, I try to advise them to take painkillers to reduce the pain because when the child is in pain there’s always discomfort… After receiving advice about the importance of completing the vaccination, they get the courage to move on and complete the clinic visits.’—IDI 2.*

### Positive CHW prototype education material feedback

Generally, the job aids shared with CHWs were well received and said to be clear, practical, and effective tools for educating community members and caregivers about vaccinations. CHWs appreciated the visual clarity, with many finding the aids easy to use and helpful in teaching vaccine types, schedules, and administration. They would incorporate the aids in daily work, particularly during household visits or training sessions. However, one concern raised was that in some images, *‘They’ll (the community members) see the injection icons and be fearful.’ (IDI 20)*. Overall, the job aids were considered valuable in enhancing understanding and promoting timely vaccinations and described by a CHW as a *‘well illustrated job aid… clear to understand…’ (IDI 14)*.

## Discussion

This study was undertaken to understand the educational needs of CHWs in effectively delivering vaccine education within their communities, in the context of the recent malaria vaccine rollout in Kenya. It also sought to assess the feasibility of integrating digital educational resources into the routine practices of CHWs. Members of the community and CHWs offered their general perceptions of childhood vaccination and the malaria vaccine, preferences for vaccine education resources, as well as general barriers faced to vaccination.

Generally, members of the community demonstrated a clear understanding of the benefits of vaccination, such as reducing the risk of illness and ensuring healthy development in children. This positive outlook is crucial as it indicates a baseline of support for vaccination efforts within the community (9). CHWs in the study were pro-vaccine, and community members placed significant trust in CHWs, viewing them as reliable sources of information due to their training and connection to health facilities. These findings reflect conclusions from numerous studies that have cited the essential role CHWs play as a resource for general vaccine knowledge, improving childhood vaccination uptake, improving care-seeking behaviour and health outcomes (19–23, 47). The community overwhelmingly prefers receiving vaccine information through in-person interactions in communal spaces or during household visits with their CHW. These settings foster a sense of familiarity and trust, making them effective platforms for vaccine education and outreach (48). This implies that health campaigns or community dialogue days and new digital interventions should be cognizant of in-person communication, utilising trusted local figures like CHWs and community leaders, and engage the community in familiar social settings (14, 31, 33, 35, 49–51).

There were instances of conflict within families about whether or not to vaccinate children. This is particularly concerning because disagreements between partners (e.g., between mothers and fathers) or extended family members can lead to delays or non-compliance with vaccination schedules. This highlights the importance of involving both parents and extended family members in educational campaigns and discussions about the benefits of vaccines to reduce discord and promote consistent vaccination practices (52–56). The community highlighted vaccine hesitancy in some families due to a combination of personal attitudes and beliefs, including fear of side effects and immediate pain caused by vaccination, mistrust of modern medicine, preference for traditional or herbal remedies, and religious beliefs. Specific myths and misinformation, such as vaccines causing infertility, lowering intelligence, or weakening the immune system, contribute to vaccine hesitancy (9–15). These misconceptions must be addressed through targeted communication and education to help shift community perceptions and increase vaccine uptake (9, 10, 14). CHWs pointed to the effectiveness of getting vaccinations themselves or in their families as helpful in debunking myths.

Community members also offered views specifically on the malaria vaccine and barriers to uptake. A comprehensive, community-centred communication strategy is necessary to educate people about the malaria vaccine. There is confusion regarding the malaria vaccine’s availability in certain regions (e.g., it is available in Migori but not in Nairobi, as priority was given to malaria endemic zones), which leads to questions about its necessity and effectiveness. This geographic disparity must be explained more effectively, clarifying where to access vaccinations to avoid misunderstandings. Due to malaria endemicity, Western Kenyan regions are malaria endemic compared to more central parts like Nairobi and Central Kenya (57). In addition, there is misinformation and skepticism about the malaria vaccine, such as believing that it is linked to family planning or harmful to children’s health. These misconceptions stem from general confusion and lack of awareness, but also misassociation with other vaccination programmes, particularly the HPV vaccine, which is also cited in other studies (12, 14). There is a lack of clear communication about the vaccine’s purpose and safety (9, 12–15). Empowering CHWs effectively to educate communities on the benefits and availability of the malaria vaccine will be key in ensuring accurate information is shared and trust is built.

However, these efforts must be coupled with an enabling environment where community structures ensure awareness building and quality care provision at the facility and county government levels. There was a clear need for better communication skills and more empathetic interactions from healthcare workers to ensure that caregivers feel supported and encouraged, rather than intimidated or shamed. The community described experiences where caregivers felt reprimanded or disrespected by healthcare providers, which discouraged them from seeking vaccination services. This is a critical issue, and is cited in other studies as contributing to long-term avoidance of health facilities and increased non-compliance (9, 58–60).

Distance from health facilities, transportation costs, and vaccine shortages emerged as significant access barriers to vaccination. These structural barriers are similarly cited in other contexts across Africa, and combined with long wait times and queues at health facilities, they discourage caregivers from taking their children for immunisation, further exacerbating the gap in vaccination coverage (3, 9, 10, 17). Efforts to improve infrastructure and reduce costs for the caregiver could alleviate some of these challenges. Literature recommends that efforts, including mobile vaccination clinics, leveraging technology, subsidised transportation, and/or partnerships with local community groups, could help reduce these challenges (3, 9, 10, 17, 19, 27, 51).

CHWs highly value in-person training sessions, especially those involving interactive visual elements. This method enhances engagement and comprehension. CHWs also rely on trusted sources such as charts, booklets, and health professionals at local health facilities and health organisations for guidance and to educate the community. While some CHWs are open to using digital tools for training, they express caution about making mistakes on work devices. However, the convenience of accessing training materials via smartphones is acknowledged, indicating that mobile learning could be beneficial to CHWs given that there is appropriate support (31, 33, 35, 36). When CHWs were presented with job aids as prototype material, the visual nature of the job aids was seen as a strong point with helpful details on vaccine types, schedules, and administration. This suggests that visual aids are practical tools that can be seamlessly integrated into community health initiatives to enhance vaccine awareness, understanding, and ensure timely vaccinations (31, 33, 61, 62).

The ‘digital gap’ or ‘digital divide’ is well documented as a challenge in other SSA countries and Kenya (63–66). Digital literacy is often discussed as many users struggle with navigating smartphones and apps in the absence of technical support. Language barriers and a lack of understanding of smartphone functionality further hinder effective use. There are also several challenges widening the digital divide, including the cost of data bundles, poor network coverage, and limited access to smartphones, particularly among older adults. Additionally, the risk of smartphone theft in some areas discourages ownership. To bridge the digital divide in Kenya, literature suggests a multifaceted approach that combines education, technology access, infrastructure improvements, and security measures. Suggestions found in the literature include training initiatives targeted at all age groups conducted in communal spaces, with a particular focus on older adults and those in rural areas who have limited exposure to technology (63–67). Others suggest simplified user interfaces, using simple or local languages with visual and audio enhancements (68). To address the high costs of data bundles and network coverage, the government and telecom companies could collaborate to offer subsidised or low-cost internet access, especially for educational and essential services (25, 28–30, 63–66, 69). Farrell (70) suggests more robust security features, such as phone tracking and remote locking (kill switches) by telecom companies and community-based initiatives to raise awareness about smartphone safety, could help discourage theft. There is an opportunity to use technology to enhance health education for both CHWs and the broader community. Appropriate training, especially in using mobile devices for health-related tasks, could help bridge these gaps.

Future research should prioritise understanding the use of digital tools for training and information dissemination, the research should examine the effectiveness of mobile-based health education for both CHWs and community members and should aim to address barriers such as digital literacy, language issues, and limited smartphone access. Furthermore, exploring how digital platforms complement traditional face-to-face training methods, such as job aids, which were seen positively by study participants, could provide valuable insights into hybrid models for health education. Investigating how CHWs incorporate training materials into their daily practices could also offer important insights into the best approaches to equipping CHWs with the necessary tools and knowledge to effectively promote vaccination. Finally, more research is needed to investigate effective communication strategies to counter misinformation and identify ways to better engage community members in educational campaigns.

## Study limitations

While qualitative assessments are generally context-specific (37, 71), and regions in southwestern Kenya will largely vary in demographics and local enablers and constraints, we recommend extending the study to other areas to see how stakeholder opinions could differ and how these insights discovered here can further strengthen tailored programmes. The study also acknowledges selection bias in that CHWs in this study are highly trained and supported, with many already using digital tools for routine data collection purposes, and thus, perceptions may differ from those of CHWs with less intensive support and baseline training. It is also acknowledged that in conducting this research, there was potential for disability bias in the interview process and FGDs. While efforts were made to ensure inclusivity and openness, there may be unintentional biases from the community members and CHWs in their responses, shaped by societal attitudes or misconceptions about vaccines. We acknowledge that a key methodological study limitation was the purposive random selection of community members to participate in the study; while FGDs require the selection of a homogenous group of strangers, we purposively selected community members to participate in the study. Additionally, having both men and women in the FGDs may have introduced male/female power dynamics that may have limited open participation in the FGD.

## Data Availability

The raw data supporting the conclusions of this article will be made available by the authors without undue reservation.

## Glossary

BCG: Bacillus Calmette-Guerin Vaccine
CHA: Community Health Assistant
CHW: Community Health Worker
CHC: Community Health Committee
CHU: Community Health Units
COVID-19: Coronavirus disease 2019
DPT: Diphtheria, Tetanus & Pertussis Vaccine
FGD: Focus Group Discussions
IDI: In-depth interviews
INT: Interviewer
KDHS: Kenya Demographic and Health Survey
WHO: World Health Organisation
LMICs: Low- and middle-income countries
MDG: Millennium Development Goals
NGO: Non-Government Organisation
RCT: Randomised Control Trial
SDG: Sustainable Development Goals
SSA: Sub-Saharan Africa
UN: United Nations
UNICEF: United Nations International Children’s Emergency Fund
RI: Routine Immunisation
HPV: Human papillomavirus
