# Supplementary material for: Strengthening vaccine uptake: a qualitative assessment of community health worker educational resource needs and community perspectives on vaccination in Western Kenya
Source: Front Public Health. 2025 Nov 14;13:1661069. doi: 10.3389/fpubh.2025.1661069 (PMC12661545; doi:10.3389/fpubh.2025.1661069)
Supplement: Supplementary file 1 [file Data_Sheet_1.docx]

**Appendix A**

Formative CHW IDIs

**Key objectives:**

- Understand views on immunisations
- Gather information on current mis/information about immunisations
- Gauge responses to vaccine education training
- Understand how health workers share/use WhatsApp

**Part 1: Introduction**

Thank you for participating in this interview.

Hello [again], my name is [x]. I’m [title] with [organizations].

In this study, we are trying to understand hesitancy around routine infant and childhood vaccinations, what social media messages would best encourage vaccination, and how to support health workers with WhatsApp learning groups. If you choose to participate in this study, I will ask a range of questions about community members' views on vaccines and your reaction to vaccine education training. Your participation will take approximately [XX] minutes.

As we ask questions, remember there are no right or wrong answers. We want you to be honest and open. Your experiences are valuable for us to understand better how to create community health messages about vaccines.

With your permission, the focus group will be audio [and video] recorded. This will help to make sure I’m getting an accurate record. The recording will be transcribed, and transcripts will only be downloaded by our research team on password-protected, encrypted computers. The recordings will be destroyed at the end of the study. Identifiable private information will be removed from the transcripts, and after such removal, the information could be used for future research studies without additional informed consent from you.

Please understand your participation is voluntary. You have the right to refuse to answer particular questions. The choice of whether to participate or not is yours alone. If you choose not to take part, you will not be affected in any way whatsoever. If you agree to participate, you may stop participating in the discussion at any time and tell me that you do not want to continue. If you do this, there will be no penalties, and you will not be prejudiced in any way. Your individual privacy and confidentiality of the information you provide will be maintained in all published and written data resulting from the study.

There are no direct benefits to participating in the study. However, the study will assist us in better understanding vaccine education. You will not receive direct payment for your participation.

The project may be published in academic journals or in conference presentations.

If you have any complaints or concerns, please feel free to contact [local study contact].

Questions/ concerns: Do you have any questions?

Is it okay to proceed with the focus group?

Do you give your permission for me to video/ audio record you?

Do you give permission for me to re-contact you to clarify information?

Do you give me permission to quote you directly without identifying you]?]

Thank you, and let’s begin!

**Part 2: Experiences with vaccines/ community vaccination campaigns**

1. Are you or have you ever been part of an immunisation or vaccination campaign as part of your job?
   1. [If yes] In your experiences, are patients or clients hesitant to receive vaccines?
      1. Which concerns do you hear about most often?
   2. [If yes] How did you address this?
2. Do you or have you worked with caregivers with children under five years of age?
   1. [If yes] What experience do you have with caregivers’ questions about vaccines?
   2. In your experience, who frequently has the most questions about vaccines? (PROMPT: Parents? Grandparents? Other caregivers?)
3. Thinking about your family, friends, patients, clients, and others in your community, what do you think causes hesitancy for routine immunisations?
4. Where do you think most community members get their information about routine immunisation?
   1. Why are those the main sources?
   2. Of those you mentioned, what are the most trusted sources for health information among community members?
5. Do you have experience with administering the malaria vaccine or responding to patients' questions about the malaria vaccine?
   1. Are community members aware of the malaria vaccine?
   2. What questions do they have about the malaria vaccine?
   3. What, if any, are common beliefs about the malaria vaccine?

**Part 3: Sharing Trustworthy Information/ Training**

1. Have you received any training about vaccines?
   1. [If yes, PROMPT] What type of training?
   2. What did it cover?
   3. Who led the training?
   4. What type of format was it (in-person, IVR, SMS, online, etc.)?
   5. Did it provide useful information for your work?
2. When you’re discussing vaccines with patients/clients, do you feel like you have the information to help them make informed decisions?
   1. [If yes] How did you obtain this information?
   2. [If no] Why not?
      1. PROMPT: Is there something you would like to know more about?
      2. What concerns do you feel least equipped to manage?
3. What do you or your colleagues do when a patient/client asks you a question you do not have information/knowledge about?
   1. What are common strategies you use to find information?
   2. What sources of information do you turn to?
4. How would you use new training materials about vaccines to train yourself?
   1. PROMPT: What type of materials would be most useful (e.g. video, infographic, written guide, etc.)
5. How do you promote vaccines in your community and/or train others?
   1. PROMPT: Can you tell us more about the others you will train?
   2. PROMPT: What type of materials are most useful (e.g. video, infographic, written guide, etc.) when you promote vaccines?
   3. PROMPT: What type of materials are most useful (e.g. video, infographic, written guide, etc.) when you train others about vaccines?

**Part 4: Reaction to Messages**

Now I am going to ask you about the content that we created about vaccines. It is just some of the content to show you as an example.

1. Would you use a video in your daily work?
2. [if yes] How would you use this in your daily work?
   1. PROMPT: Would you share this information with patients/clients? If so, how?
3. What do you think about this job aid? [show [Posters](https://show.pics.io/preview/63c1c40f26ed9d0014b22f12)]
   1. PROMPT: Was it easy to understand? Was it clear?
4. What did you learn from this job aid?
   1. PROMPT: Will the content of this job aid be helpful to teach about vaccines?
5. Is there anything you found confusing about this job aid?
   1. PROMPT: Was any of the information confusing to you?
   2. PROMPT: Was any of the language confusing to you?
   3. PROMPT: Were any of the images confusing to you?
6. How would you use this in your daily work?
   1. PROMPT: Would you share this information with patients/clients? If so, how?

**Part 5: Conclusion**

Thank you so much for sharing your views and providing valuable input. Our next steps are to gather all your feedback and update what we need to. We will then continue developing the rest of the messages.

Appendix B

Focus Group Discussions

**Key objectives:**

- Understanding views on immunisations
- Gather information on current mis/information about immunisations
- Gauge responses to vaccine related social media messages
- Understand what vaccine/ health messages people view as trustworthy

**Part 1: Introduction**

Thank you for participating in this focus group.

Hello [again], my name is [x]. I’m [title] with [organization].

In this study, we are trying to understand hesitancy around routine infant and childhood vaccinations. If you choose to participate in this study, I will ask a range of questions about community members' views on vaccines and your reaction to social media messages. Your participation will take approximately [XX] minutes.

As we ask questions, remember that there are no right or wrong answers. We want you to be honest and open. Your experiences are valuable for us to better understand how to create community health messages.

With your permission, the focus group will be audio [and video] recorded. This will help to make sure I’m getting an accurate record. The recording will be transcribed and transcripts will only be downloaded by our research team on password protected, encrypted computers. The recordings will be destroyed at the end of the study. Identifiable private information will be removed from the transcripts and, after such removal, the information could be used for future research studies without additional informed consent from you.

Please understand your participation is voluntary. You have the right to refuse to answer particular questions. The choice of whether to participate or not, is yours alone. If you choose not to take part, you will not be affected in any way whatsoever. If you agree to participate, you may stop participating in the discussion at any time and tell me that you do not want to continue. If you do this, there will be no penalties and you will not be prejudiced in any way. Your individual privacy and confidentiality of the information you provide will be maintained in all published and written data resulting from the study. We also ask that you respect your fellow participants’ confidentiality and opinions throughout this discussion.

There are no direct benefits to participating in the study, however, the study will assist us to better understand vaccine education. You will not receive direct payment for your participation.

The project may be published in academic journals or in conference presentations.

If you have any complaints or concerns please feel free to contact [local study contact].

Questions/ concerns: Do you have any questions?

Is it okay to proceed with the focus group?

Do you give your permission for me to video/ audio record you?

Do you give permission for me to re-contact you to clarify information?

Do you give me permission to quote you directly without identifying you]?]

Thank you and let’s begin!

**Part 2: Views on Childhood immunisations**

1. What vaccines are you familiar with for your children?
2. Do you have any concerns about vaccinating your children?
   1. [If yes] What are some of your concerns about vaccines for your children?
3. Have you ever faced any challenges when trying to have your child vaccinated?
   1. [if yes] Can you tell me more about that experience? What happened? What made it challenging?
   2. Were you able to address the challenges? How did you overcome those challenges?
4. Thinking about your family, friends, and others in your community, what do you think causes people to be hesitant to vaccinate their children?
5. Where do you go most of the time for vaccines?
6. What has helped you [or would help you] feel more comfortable about bringing your children in for receiving vaccines?
7. Thinking about your family, friends, and others in your community, who makes decisions about vaccinating children usually? (i.e.- Parents, grandparents, other family members).

**Part 3: Views on the malaria vaccine**

1. What do you know about the malaria vaccine?
   1. PROMPT: Who is eligible for the malaria vaccine?
   2. PROMPT: What have you heard about the malaria vaccine?

**Part 4: Misinformation & Trustworthy Information**

1. In what way do you seek health or vaccine information in the community?
   1. PROMPT: What sources of information do you turn to?
2. Where do community members obtain information about vaccines?
   1. Why are those the main sources?
   2. Of those you mentioned, what are the most trusted sources for health information among community members?
      1. Do they trust info from radio, TV, SMS, or social media?
3. Do you feel people think of CHWs as a useful source of information for vaccine information?
4. Thinking about your family, friends, and others in your community, do most people have access to smartphones?
   1. Are there any barriers that would prevent you or others in your community from accessing digital materials?
   2. PROMPT: lack of access to devices or data, a limited amount of time available to take trainings), limited literacy?
5. Do you use WhatsApp?
   1. Have you ever shared any information you’ve learned about your health on WhatsApp?
   2. [If yes, PROMPT] What type of information?
   3. [If no, PROMPT] Why not?
      1. Are there other social media sites that you share health information on? What sites? (PROMPT: SMS, USSD, Facebook Messenger?)

**Part 5: Conclusion**

Thank you so much for sharing your views and providing valuable input. Our next steps are to gather all your feedback and update what we need to. We will then continue developing the rest of the messages.
